# New insights into the testicular tropism of porcine reproductive and respiratory syndrome virus

**DOI:** 10.1128/spectrum.02964-24

**Published:** 2025-02-19

**Authors:** Kassandra Durazo-Martínez, Fernando A. Osorio, Gustavo Delhon, Jesús Hernández, Hiep L. X. Vu

**Affiliations:** 1 Nebraska Center for Virology, University of Nebraska-Lincoln, Lincoln, Nebraska, USA; 2 Department of Animal Science, University of Nebraska-Lincoln, Lincoln, Nebraska, USA; 3 School of Veterinary Medicine and Biomedical Sciences, University of Nebraska-Lincoln, Lincoln, Nebraska, USA; 4 Laboratorio de Inmunología, Centro de Investigación en Alimentación y Desarrollo A.C. (CIAD, A.C.), Hermosillo, Sonora, Mexico; Oklahoma State University College of Veterinary Medicine, Stillwater, Oklahoma, USA

**Keywords:** PRRSV, spermatogonia stem cells, testicular tropism, swine viruses

## Abstract

Porcine reproductive and respiratory syndrome virus (PRRSV) has a restricted host specificity, primarily infecting porcine macrophages. Notably, an exception to such macrophage-restricted tropism has been observed in sexually active boars, where the virus infects and induces apoptosis in the germinal epithelium, resulting in viral dissemination in the ejaculate. Whether this phenomenon occurs in prepubertal animals remains unclear. In this study, we isolated spermatogonia stem cells (SSCs) from neonatal pigs and cultured them *in vitro*. These SSC cultures formed morula-like colonies, exhibited alkaline phosphatase activity—a characteristic of stem cells—and expressed protein gene product 9.5, a marker of SSCs. Notably, the SSC cultures supported PRRSV replication with kinetics similar to that observed in porcine alveolar macrophages. To assess the testicular tropism of PRRSV in prepuberal animals, 28-day-old male pigs were infected with a virulent PRRSV strain. Testicular tissues were sequentially analyzed using a combination of *in situ* hybridization for PRRSV RNA and immunohistochemistry for specific cellular markers. Unlike in sexually active boars, PRRSV did not infect the spermatogonia cells within the seminiferous tubules of prepubertal pigs. Instead, the virus primarily infected macrophages and myoid cells located in the interstitium and peritubular areas. It appeared that the anatomical separation of spermatogonia from the basal membrane of the seminiferous tubules in prepubertal pigs prevents these cells from being infected by PRRSV. Overall, our findings offer valuable insights into the age-dependent testicular tropism of PRRSV.

**IMPORTANCE:**

Contaminated boar semen used in artificial insemination has significantly contributed to the global spread of porcine reproductive and respiratory syndrome virus (PRRSV), a virus that typically infects only cells within the monocyte and macrophage lineages. Our study reveals that spermatogonia stem cells (SSCs) from neonatal piglets are also susceptible to PRRSV, suggesting that non-macrophage cells can be infected by the virus. However, despite this susceptibility, PRRSV-infected cells were not found in the seminiferous tubules of prepubertal male pigs inoculated with a virulent PRRSV strain. This contrasts with sexually mature boars, where PRRSV-infected cells were prominently observed within the seminiferous tubules. The discrepancy is likely due to anatomical differences between the seminiferous tubules of sexually mature boars and prepubertal pigs. These findings provide new insights into PRRSV pathogenesis. Additionally, the *ex vivo* SSC culture provides a valuable model for identifying new viral receptors necessary for PRRSV infection and for investigating the virus’s impact on spermatogenesis.

## INTRODUCTION

Porcine reproductive and respiratory syndrome virus (PRRSV) is a single-stranded positive-sense RNA virus classified under the order *Nidovirales*, family *Arteriviridae*, and genus *Betaarteriviruses*. First reported in the United States in 1987 and later in Europe in 1990 (1, 2), the virus has since spread worldwide and become one of the most economically devastating infectious diseases in swine. PRRSV infection of pregnant sows often causes reproductive issues, including increased abortions and stillbirths (3). In boars, PRRSV infection results in decreased fertility and semen quality (4). Notably, it has been reported that the virus persists in the cellular fraction of ejaculates from infected animals for extended periods (5). Contaminated semen used in artificial insemination is a major factor in the global spread of the disease (6).

In sexually mature boars experimentally infected with PRRSV, the virus is transported to the testes by infected, migrating macrophages, leading to infection of testicular macrophages and germinal epithelium cells (7–10). PRRSV infection of testicular tissue causes the formation of multinucleated giant cells and significant germ cell depletion and death by apoptosis, resulting in reduced semen quality (6, 7). In the semen of infected boars, PRRSV genome is consistently detected in the cellular fraction, rather than in the seminal plasma, specifically within cells identified as macrophages and immature germ cells (7, 10).

Multiple cellular receptors have been demonstrated to be involved in PRRSV infection (11). CD163 is considered the primary receptor for PRRSV infection of swine macrophages (12). Transfection of non-susceptible cell lines, such as baby hamster kidney 21 (BHK-21) and porcine kidney 15 (PK-15) cells with CD163 derived from different species—including swine, human, monkey, dog, and mouse—renders these cells susceptible to PRRSV infection (12). Conversely, gene-edited pigs that lack all or part of CD163 are completely resistant to PRRSV infection (13–16). While this demonstrates the critical role of CD163, evidence suggests that it may not be the sole determinant of susceptibility. Although CD163 from humans and mice can confer susceptibility to other cell lines, the original human and mouse cell lines from which CD163 was cloned, remain non-susceptible (12). This indicates that additional unidentified cellular factors are required for productive PRRSV infection, which might be present in cell lines like BHK-21 and PK-15 but absent in human and mouse macrophages (12). Vimentin has also been identified as a potential receptor for PRRSV (17). Transfection of cell lines such as BHK-21 and Crandell-Rees feline kidney cells, which do not express CD163, with vimentin conferred susceptibility to PRRSV infection. On the other hand, blocking vimentin in MARC-145 cells with a polyclonal antibody prior to PRRSV inoculation completely prevented infection (17). Similarly, ectopic expression of CD151 in BHK-21 cells is sufficient to render them susceptible to PRRSV, while pretreating MARC-145 cells with an anti-CD151 antibody completely blocked PRRSV infection (18). This evidence indicates that PRRSV can still infect cells lacking CD163 if other factors, such as vimentin and CD151, are present.

Although testicular tropism of PRRSV has been observed in multiple studies (7–9), the exact identification of susceptible cells within testicular tissue remains poorly understood. Moreover, it is unclear whether PRRSV uses CD163 or alternative receptors to infect these cells. Additionally, whether PRRSV can infect testicular cells in young pigs has yet to be determined.

In this study, we successfully cultured spermatogonia stem cells (SSCs) isolated from neonatal piglets and demonstrated their susceptibility to PRRSV infection. Additionally, we infected prepubertal male pigs with a virulent PRRSV strain and examined the distribution of virus-infected cells in testicular tissues collected at various time points post-infection. In prepubertal pigs, PRRSV-infected cells were primarily found in the interstitium and at the periphery of the seminiferous tubules but not in the germinal center where SSCs reside. Collectively, the results of this study provide novel insights into the testicular tropism of PRRSV.

## MATERIALS AND METHODS

### Medium, cells, viruses, and antibodies

The MARC-145 cell line, cloned from MA-104 cells (19), was provided by Dr. Miller at the USDA Agricultural Research Service National Animal Disease Center. The cells were cultured in low glucose, low bicarbonate Dulbecco’s modified Eagle medium (DMEM). Porcine alveolar macrophages (PAMs) used here were obtained from lung lavage of pigs between 4 and 8 weeks old and cultured in RPMI-1640 medium. All media were supplemented with 10% fetal bovine serum (FBS) and antibiotics (100 units/mL penicillin, 100 µg/mL streptomycin). Mouse monoclonal antibody specific to PRRSV nucleocapsid (N) clone SDOW17 was purchased from National Veterinary Services Laboratories, (Ames, IA, USA). Mouse anti-N antibody conjugated with fluorescein isothiocyanate (FITC) (clone SR-30) was obtained from Rural Tech Inc. Mouse antibody against protein gene product 9.5 (PGP9.5; clone 346CT2.5.1) was obtained from Abcam (Waltham, MA, USA). Antibodies specific for Müllerian inhibiting substance (MIS; clone 1A4) and alpha-smooth muscle actin (ASMA; clone B11) were purchased from Santa Cruz Biotechnology (Dallas, TX, USA). The anti-porcine CD163 (clone 2A10/11) and anti-CD172a (clone BLH7) antibodies were obtained from Thermo Fisher Scientific (Walthman, MA, USA). The PRRSV strain FL12 was recovered from a full-length cDNA infectious clone (20).

### Preparation of SSCs

SSC cultures were established following the protocol described previously (21). Briefly, testes were collected from 1- or 2-day-old piglets at the University of Nebraska-Lincoln (UNL) Swine Research farm after routine castration and transported to the laboratory in ice-cold DMEM supplemented with 100 units/mL penicillin, 100 µg/mL streptomycin, and 2.5 µg/mL amphotericin B. Upon arrival, the testes were decapsulated and immersed in 70% ethanol for 1 minute, followed by three washes in the medium. The tissue was minced and sequentially incubated with 0.1% (wt/vol) type IV collagenase (Sigma-Aldrich) for 15 minutes, 0.1% (wt/vol) hyaluronidase (Sigma-Aldrich) for 10 minutes, and 0.25% trypsin-EDTA (Thermo Fisher Scientific) for 10 minutes, all at 37°C. The resulting cell suspension was filtered through a 70 µm nylon mesh to remove Leydig and myoid cells, and erythrocytes were removed by incubating the cells in 1× red blood cell lysis buffer (Invitrogen) for 15 minutes at room temperature. The cells were then resuspended in a complete DMEM-F12 medium (a 1:1 mixture of Ham’s-F12 and DMEM) supplemented with 10% FBS, 100 units/mL penicillin, 100 µg/mL streptomycin, and 2.5 µg/mL amphotericin B. To enrich SSCs, the cell suspension was plated on 0.1% gelatin-coated Petri dishes and incubated overnight at 37°C in a CO_2_ incubator. Loosely adherent cells were collected, re-seeded onto gelatin-coated dishes, and incubated again overnight at 37°C. The enriched cell population was then plated on Sertoli feeder cells and cultured at 37°C with 5% CO_2_ for 3 weeks. SSCs were collected by gently rocking the plate to dislodge cells loosely adhered to the surface.

### Preparation of Sertoli cells

Sertoli cells were cultured from the dissociated testicular cell population of neonatal pigs, following a previously described protocol with minor modifications (22). The cell suspension was filtered through a 500 µm stainless steel mesh and resuspended in a solution of 1 M glycine and 2 mM EDTA (both from Sigma-Aldrich) for 10 minutes at room temperature. After washing with phosphate-buffered saline (PBS, pH 7.4), the cells were resuspended in Ham’s-F12 medium supplemented with 0.166nM retinoic acid and 1:100 insulin-transferrin selenium (Sigma-Aldrich), seeded into Petri dishes, and incubated at 37°C with 5% CO_2_. On day 3, the medium was replaced, and the cells were treated with 10 mM tris-hydroxymethyl-aminomethane hydrochloride (TRIS; Sigma-Aldrich) for 10 minutes at 37°C to eliminate residual germ cells. Following TRIS treatment, the cells were washed with PBS and replenished with Ham’s-F12 supplemented with retinoic acid and insulin-transferrin selenium. The cultures were maintained at 37°C with 5% CO_2_ for 1 week before 10% FBS was added. Once confluent, the cells were trypsinized and transferred to new Petri dishes to form feeder layers for SSC culture.

### Phenotypic characterization of SSCs

For the assessment of alkaline phosphatase (AP) activity in SSCs, once colonies were visible in culture, the cells were gently washed twice with PBS and incubated with the AP substrate (ImmPACT Vector Red; Vector Laboratories) for 20 minutes in the dark. After incubation, the cultures were washed again twice with PBS and observed under a microscope.

To detect the expression of the PGP9.5 marker, harvested SSCs were washed with PBS, fixed in 4% paraformaldehyde (PFA), and cytospun onto glass slides at a density of 2 × 10^5^ cells per spot. The cells were then permeabilized with 0.1% Triton X-100 and blocked for non-specific binding using CAS-Block universal blocking agent (Thermo Fisher Scientific). After blocking, the cells were incubated for 1 hour at room temperature with the anti-PGP9.5 antibody followed by incubation with Alexa Fluor 594-conjugated anti-mouse IgG. Nuclei were stained with DAPI, and the slides were mounted with ProLong Glass Antifade Medium (Invitrogen), cured for 24 hours, and observed under an inverted microscope (Nikon Eclipse TE2000-U).

To assess the frequency of cells expressing the PGP9.5 marker, SSCs from various culture batches were washed with fluorescence-activated cell sorting (FACS) buffer (1× PBS with 4% FBS), fixed in 4% PFA, and permeabilized with 0.1% Triton X-100. The cells were then incubated for 1 hour at room temperature with an anti-PGP9.5 antibody directly conjugated to Alexa Fluor 647. The cells were analyzed using the CytoFLEX instrument, and data were processed with FlowJo software.

### Assess the susceptibility of SSCs to PRRSV infection

To assess the susceptibility of SSCs to PRRSV infection, harvested SSCs were suspended in a complete DMEM medium at a density of 1 × 10^6^ cells/mL and cultured in flow cytometry tubes. The cells were inoculated with the PRRSV strain FL12 at multiplicity of infection (MOI) of 0.1 or 10. At 12 hours post-infection (hpi), the cells were washed twice with PBS, fixed with 4% PFA for 20 minutes at room temperature, and cytospun onto Superfrost/Plus slides at a density of 2 × 10^5^ cells per spot.

For virus detection, two assays were employed: *in situ* hybridization (ISH) and indirect immunofluorescent assay (IFA). ISH was performed using RNAscope technology (Advanced Cell Diagnostics, ACD), following the manufacturer’s instructions. A ready-to-use RNA probe specific to the PRRSV open reading frame 7 (ORF7, catalog no. 870971) was used. Positive cells were visualized using the RNAscope 2.5 HD brown detection kit (ACD). The slides were counterstained with hematoxylin, then mounted and examined under a light microscope. For IFA, cells were permeabilized with 0.1% Triton X-100 for 10 minutes at room temperature before incubating with a monoclonal antibody specific to the viral N protein, followed by a secondary antibody, goat anti-mouse IgG conjugated to Alexa Fluor 488 (Invitrogen). Nuclei were stained with DAPI, and the slides were mounted with ProLong Glass Antifade Medium (Invitrogen), cured for 24 hours, and observed under an inverted microscope (Nikon Eclipse TE2000-U).

To quantify the frequency of SSCs infected with PRRSV, after fixation and permeabilization, the cells were incubated with an antibody specific to the viral N protein (clone SR-30, labeled with FITC) and the anti-PGP9.5 antibody (labeled with Alexa Fluor 647). After incubation, the cells were analyzed using a CytoFLEX instrument, and data were processed with FlowJo software.

### Multi-step growth curve of PRRSV

To determine replication kinetics, 5 × 10^5^ MARC-145, PAM, and SSCs were inoculated with PRRSV strain FL12 at an MOI of 0.1. After a 1 hour adsorption period, cells were washed three times with culture medium and replenished with fresh complete medium. At various time points post-infection, culture medium samples were collected, and virus yield was assessed by titration on MARC-145 cells. Virus titers were expressed as log10 tissue culture infectious dose 50 (TCID_50_) per mL.

### Animal experiment

Twelve uncastrated male piglets, 21 days old, were obtained from the UNL Swine Research Farm, which was confirmed to be free of PRRSV. The pigs were housed in a biosafety level 2 animal facility at UNL with free access to food and water. After a 1week adaptation period, the pigs were intramuscularly infected with 1 × 10^5^ TCID_50_ of the PRRSV strain FL12.

At 1 day post-infection (dpi), serum samples were collected from all inoculated animals to measure viral loads using real-time RT-PCR. After that, three animals were randomly selected and euthanized at 1, 3, 8, and 15 dpi, respectively, using a fatal dose of sodium pentobarbital. Their testes were collected and fixed in 10% neutral buffered formalin. Testes collected from three PRRSV-free pigs of approximately the same age, obtained from a separate study, were used as non-infected controls. The tissues were then paraffin-embedded and sectioned following standard operating procedures at the Nebraska Veterinary Diagnostic Center. Tissue sections (5 µm thick) were stained with hematoxylin and eosin (H&E) to examine histological changes. Additional tissue sections were used for ISH to detect virus-infected cells and immunohistochemistry (IHC) to detect cellular markers.

ISH was performed using RNAscope technology (ACD), following the manufacturer’s instructions. An RNA probe specific to the PRRSV ORF7 was used (23). Positive cells were visualized using the RNAscope 2.5 HD brown detection kit (ACD). The slides were counterstained with hematoxylin, then mounted and examined under a light microscope.

For dual ISH-IHC staining, after developing the ISH signal, the slides were blocked with CAS-Block and incubated overnight at 4°C with one of the following primary antibodies: PGP9.5, MIS, ASMA, CD163, or CD172a. The slides were then treated with the ImmPRESS-AP horse anti-mouse IgG polymer detection kit, and the AP signal was developed using ImmPACT Vector Red (Vector Laboratories). Finally, the slides were counterstained with hematoxylin and prepared for imaging.

## RESULTS

### Isolation and culture of SSCs

**Fig 1:**
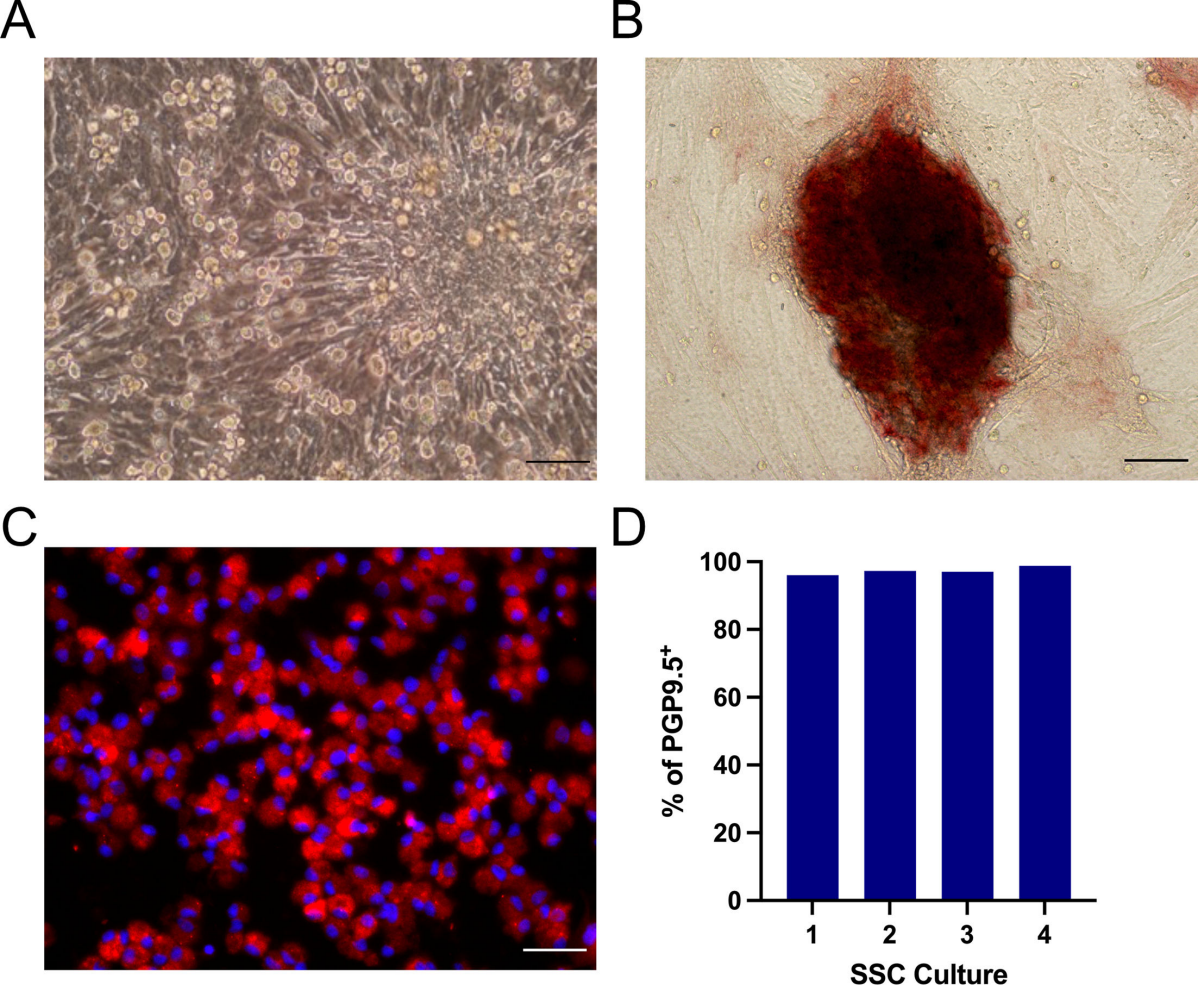
Phenotypic characterization of SSCs cultured *ex vivo*. (**A**) Phase-contrast image of an 18-day-old SSC culture. Clusters of SSCs and an SSC colony displaying the characteristic morula-like structure can be observed. (**B**) Representative image of 10-day-old cultured cells exposed to AP substrate with red indicating positive AP activity. (**C**) Representative image showing the expression of the PGP9.5 marker. PGP9.5 is visualized in red, while cell nuclei are stained with DAPI in blue. (**D**) Percentage of PGP9.5-positive cells across four different culture batches, as determined by flow cytometry. Images are representative of at least three independent experiments. Scale bar: 50 µm.

SSCs were enriched from the cell population dissociated from neonatal porcine testes by differential plating on gelatin-coated Petri dishes. Subsequently, the enriched SSC population was plated on top of Sertoli cell monolayers, which served as the feeder cells. Around 14–18 days of culture, distinctive cell clusters resembling the morula-like colonies were observed on top of the Sertoli cell layer (Fig. 1A). These colonies were similar to those observed in SSC cultures from other mammalian species (24–26). AP, a key marker of pluripotent stem cells (27), was detected only in the morula-like colonies when the SSC cultures were exposed to an AP substrate with no positive signals observed in the areas containing Sertoli cells (Fig. 1B). PGP9.5, also known as ubiquitin carboxyl-terminal hydrolase L1, a specific marker of SSCs in the pig testes (28), was readily detected in the harvested SSCs by IFA (Fig. 1C). Quantitatively, over 90% of the harvested SSCs from different culture batches expressed the PGP9.5 marker (Fig. 1D). Overall, the formation of morula-like colonies, along with AP activity and PGP9.5 expression, confirms that SSCs were successfully isolated and enriched from the testicular cells of neonatal pigs.

### Susceptibility of SSCs to PRRSV infection

**Fig 2:**
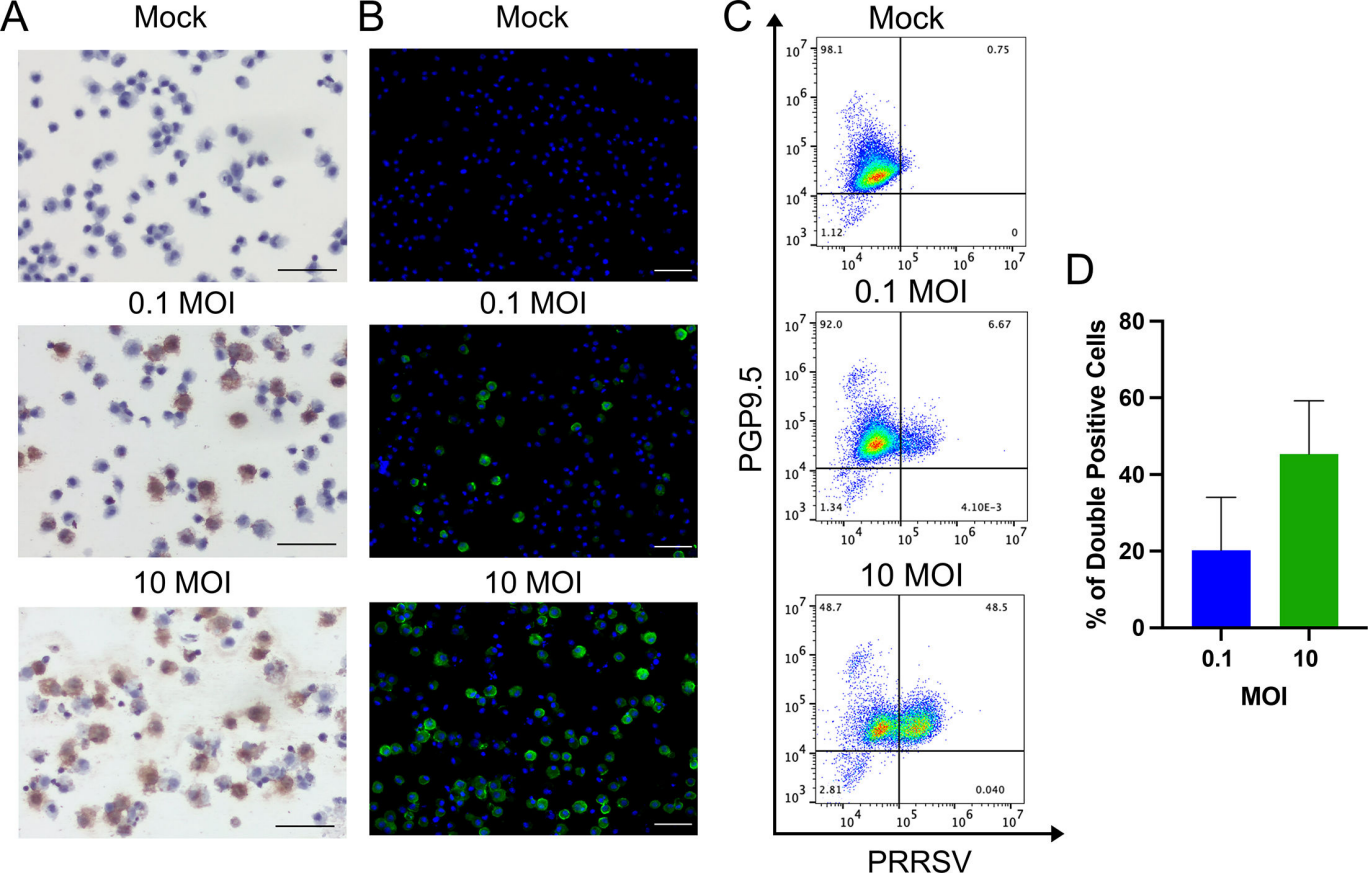
Susceptibility of SSCs to PRRSV infection. SSCs were cultured in suspension and inoculated with PRRSV strain FL12 at an MOI of 0.1 or 10. At 12 hpi, the cells were collected and processed for ISH to detect viral mRNA or for an IFA to detect viral N protein. Cells were also analyzed by flow cytometry to quantify the frequency of virus-infected cells. (**A**) Representative images from the ISH assay detecting PRRSV mRNA. Viral mRNA is shown in brown, and cell nuclei are counterstained with hematoxylin (blue). (**B**) Representative images from the IFA detecting viral N protein. Viral N is shown in green, while nuclei are stained with DAPI (blue). (**C**) Representative flow cytometry charts of infected SSCs stained with antibodies against PRRSV N protein and the PGP9.5 marker. (**D**) The frequencies of cells double-positive for PRRSV N protein and the PGP9.5 marker. Data are expressed as the mean ± standard deviation from three independent experiments. Scale bar: 50 µm.

SSCs were harvested approximately 14 days after co-culture with Sertoli cells and then infected with the PRRSV strain FL12 at MOI of 0.1 and 10. At 12 hpi, virus-infected cells were detected using two assays: ISH with a probe targeting the viral N transcripts and IFA with an antibody specific to the viral N protein. Numerous ISH-positive and IFA-positive cells were observed, with higher numbers of positive cells in cultures inoculated with 10 MOI compared to 0.1 MOI (Fig. 2A and B). To quantify the frequency of virus-infected cells, we simultaneously stained the cells with antibodies specific to the viral N protein and the PGP9.5 marker. At 0.1 MOI, approximately 20% of the cells were double-positive for both markers, and this pattern increased to over 40% at 10 MOI (Fig. 2C and D). These findings demonstrate that SSCs cultured *ex vivo* are highly susceptible to PRRSV.

### Replication kinetics of PRRSV in SSCs

**Fig 3:**
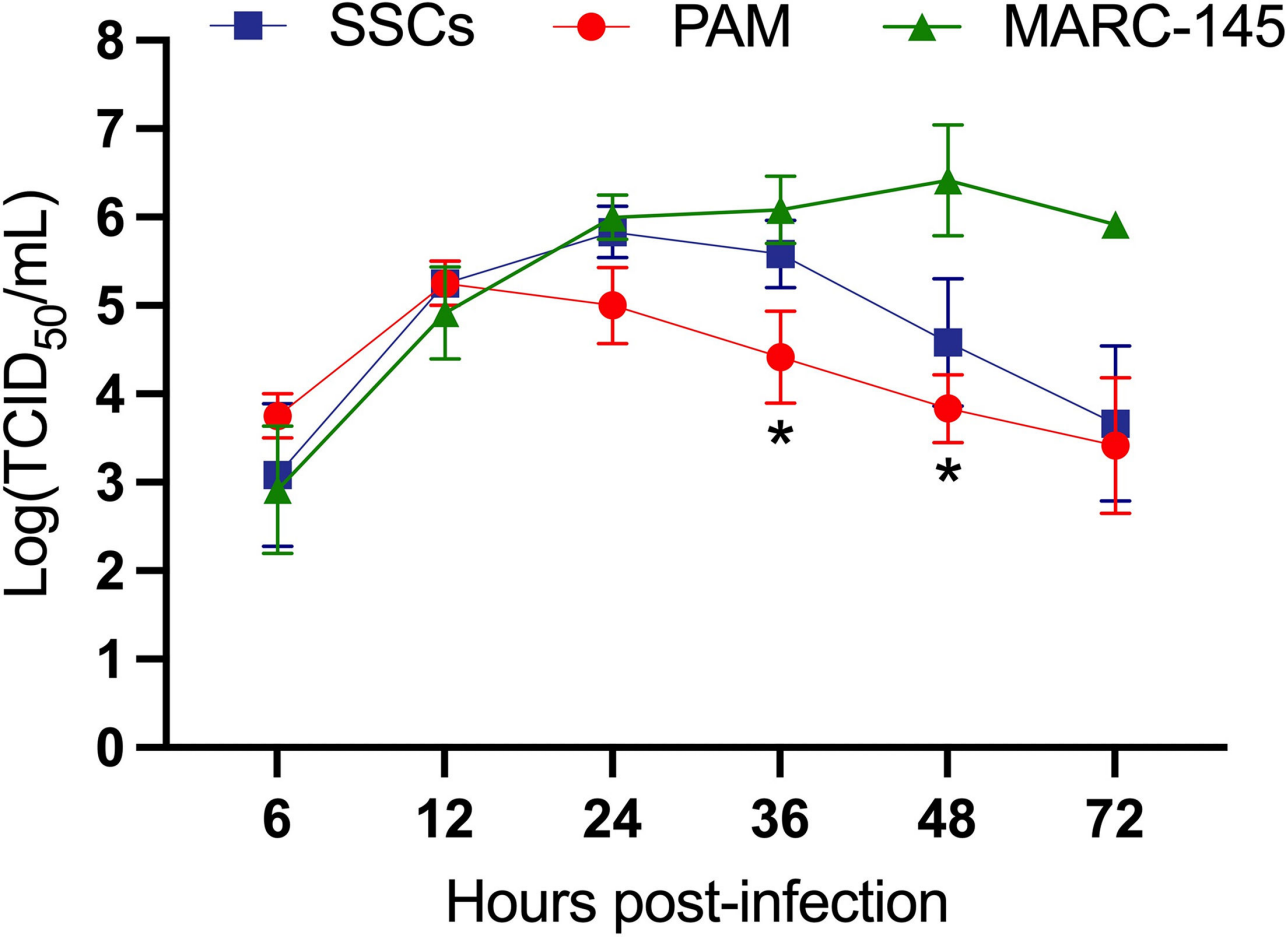
Multiple-step growth curve. Data are expressed as the mean ± standard deviation from three independent experiments. An asterisk (*) indicates a statistically significant difference compared to the virus titers in MARC-145 cells. *P < 0.05.

Next, we evaluated the replication kinetics of PRRSV in SSCs and compared them to the virus’s replication in PAMs, the primary target of PRRSV (29), and MARC-145, a monkey cell line permissive to the virus. In SSCs, progeny virus was first detected at 6 hpi, rapidly increased after 12 hpi, peaked at 24 hpi, and then gradually declined (Fig. 3). In PAMs, virus yield peaked at 12 hpi and started to decrease afterward. In MARC-145 cells, the highest virus titers were observed at 48 hpi, followed by a slight decline by 72 hpi. By 24 hpi, virus titers in SSCs and MARC-145 cells were similar, while PAMs had lower titers. At 36 and 48 hpi, the virus titers in PAMs were significantly lower than in MARC-145 cells. Although virus titers in SSCs were higher than in PAMs at 24, 36, and 48 hpi, these differences were not statistically significant. Overall, SSCs supported PRRSV replication with kinetics similar to those observed in PAMs.

### Testicular tropism of PRRSV in prepubertal pigs

**Fig 4:**
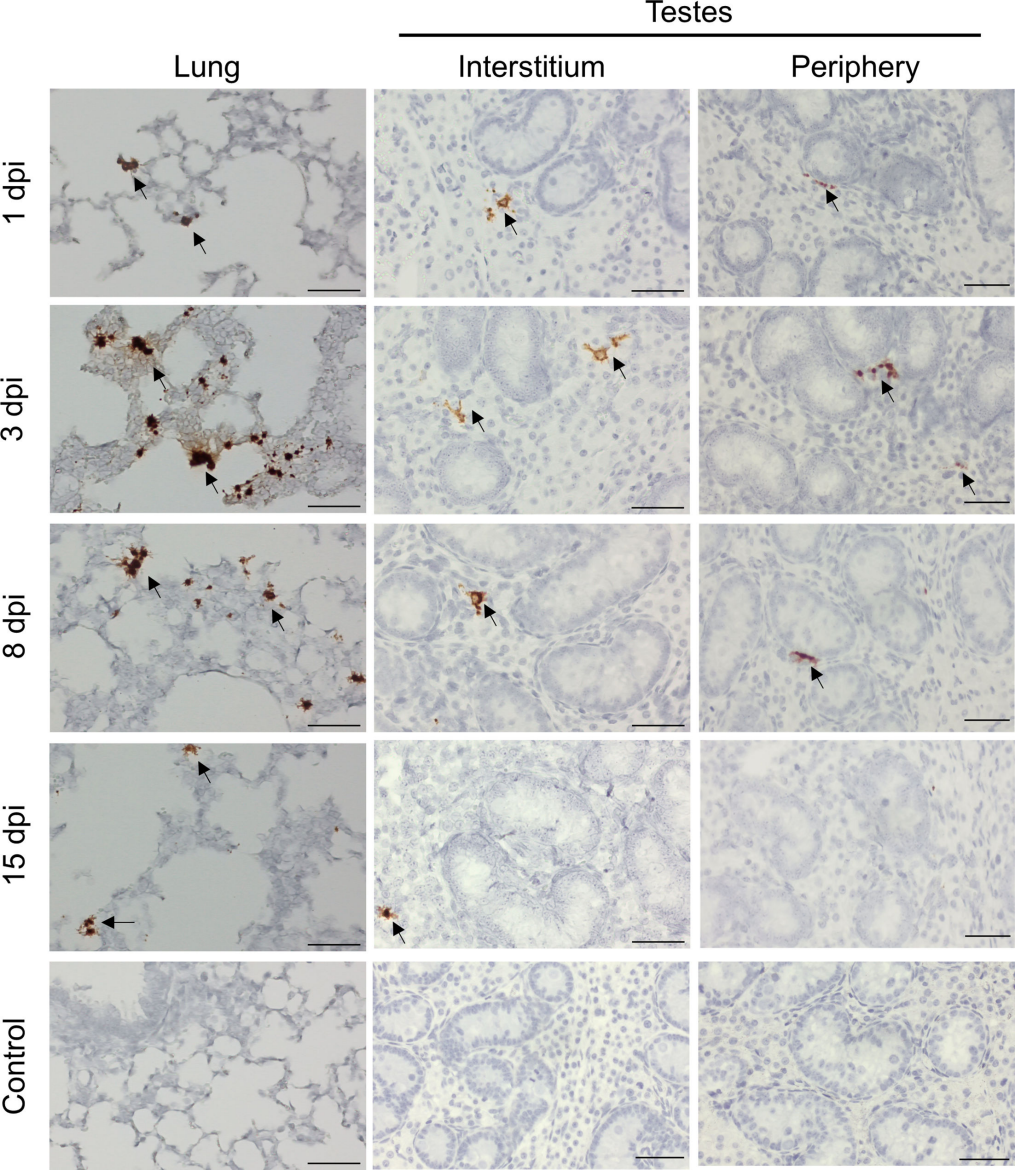
Localization of PRRSV-infected cells in lung and testicular tissue of prepubertal pigs. Uncastrated pigs were inoculated intramuscularly with PRRSV strain FL12 at a dose of 1 × 10⁵ TCID_50_. At indicated days post-infection (dpi), lungs and testes were collected and processed for ISH to detect viral mRNA transcripts using a probe specific to ORF7. Brown staining indicates virus-infected cells (arrow), with hematoxylin (blue) used as a counterstain. Scale bar: 50 µm.

To investigate PRRSV testicular tropism in sexually immature animals, we infected 12 uncastrated piglets at 28 days old with the PRRSV strain FL12. Viral genomic RNA was detected in the blood of all infected piglets from day 1 through day 15 post-infection, confirming successful infection (Table 1). At various time points, groups of three randomly selected infected piglets were euthanized, and their lung and testicular tissues were collected to detect virus-infected cells using ISH assay. Numerous virus-infected cells were observed in lung sections of infected pigs (Fig. 4), further confirming successful infection and the specificity of the ISH assay. In testes, virus-infected cells were consistently found in the interstitium from day 1 to day 15, though the signal decreased toward the end of the experiment, corresponding to the reduction in viremia (Fig. 4). Virus-infected cells were also observed at the periphery of the seminiferous tubules from 1 dpi to 8 dpi but were rarely observed at 15 dpi (Fig. 4). Importantly, no virus-infected cells were detected within the germinal epithelium at any time point, indicating that the virus does not infect the germinal epithelia of prepubertal pigs.

**TABLE 1:** Viral loads in serum samples determined by RT-PCR

Pig ID number	Days post-infection (CT value)				
	0	1	3	8	15
1	Undetermined	18.2			
2	Undetermined	17.8			
3	Undetermined	20.5			
4	Undetermined	20.3	19.6		
5	Undetermined	18.1	14.0		
6	Undetermined	18.5	17.0		
7	Undetermined	14.9		13.7	
8	Undetermined	18.4		21.6	
9	Undetermined	19.0		14.5	
10	Undetermined	17.5			21.8
11	Undetermined	18.6			27.4
12	Undetermined	18.6			29.6

### Histological changes in the testes

**Fig 5:**
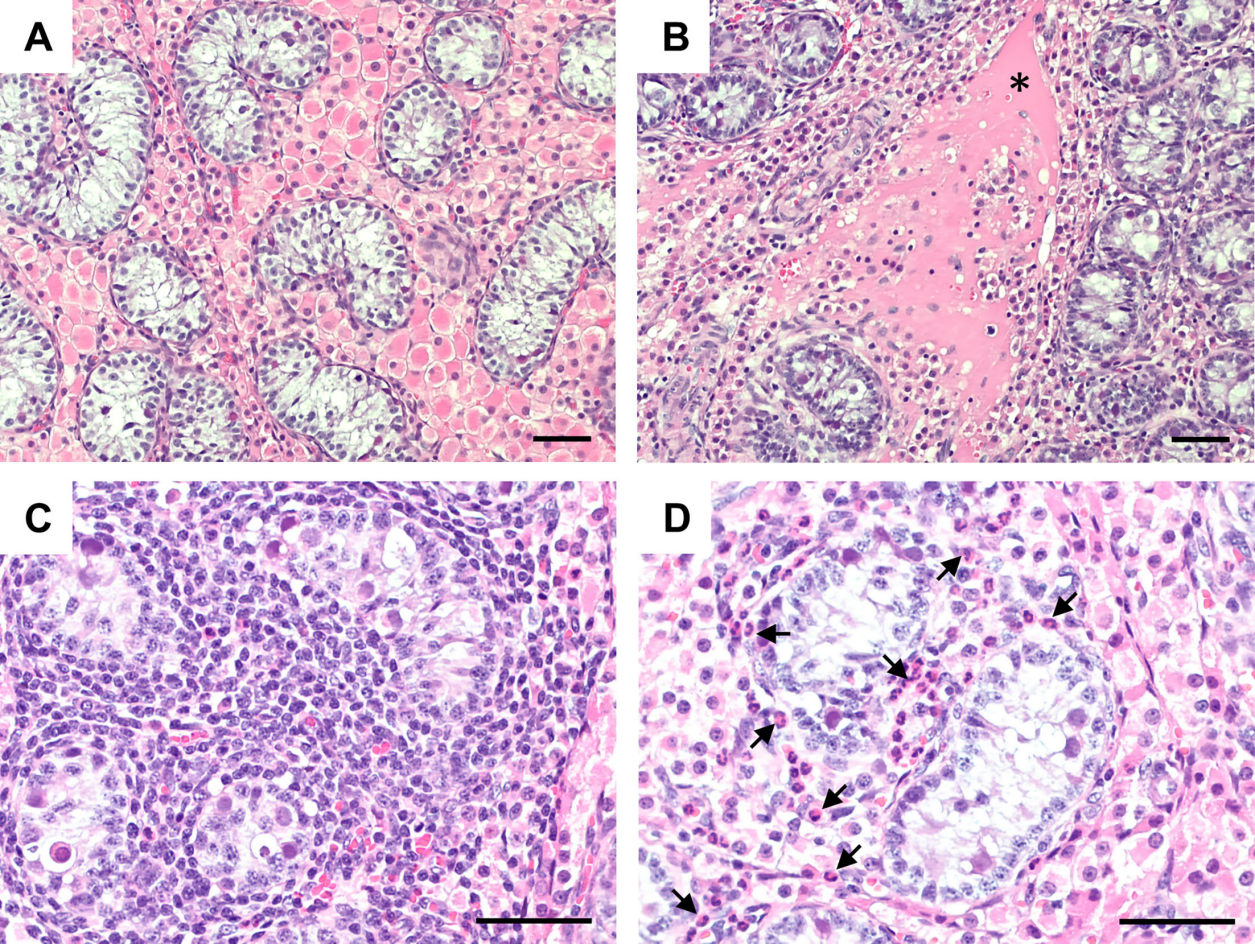
H&E staining of prepuberal pig testes. (**A**) Representative image showing a section of normal, uninfected testis. (**B–D**) Images showing histological changes in the testis at 15 days after infection with the PRRSV strain FL12. (**B**) An area with interstitial edema, marked by the asterisk. (**C**) An area with focal interstitium inflammatory infiltration. (**D**) An area with focal interstitium infiltration of neutrophils, indicated by arrows. Note that not every neutrophils are marked. Scale bar: 50 µm.

Given the detection of virus-infected cells in the testes, we next examined the histological changes in the tissue. Mild histological changes were observed in the testes collected at 15 dpi. Among the three pigs euthanized at 15 dpi, interstitial edema was present in all three pigs (Fig. 5A), focal interstitial inflammatory infiltration of mononuclear cells was observed in two pigs (Fig. 5B), and focal interstitial infiltration of neutrophils was seen in one pig (Fig. 5C). No significant histological changes were detected in tissue sections of testes collected at earlier time points, despite the presence of virus-infected cells in those tissues.

### Putative cell types infected by PRRSV in prepubertal pig testes

**Fig 6:**
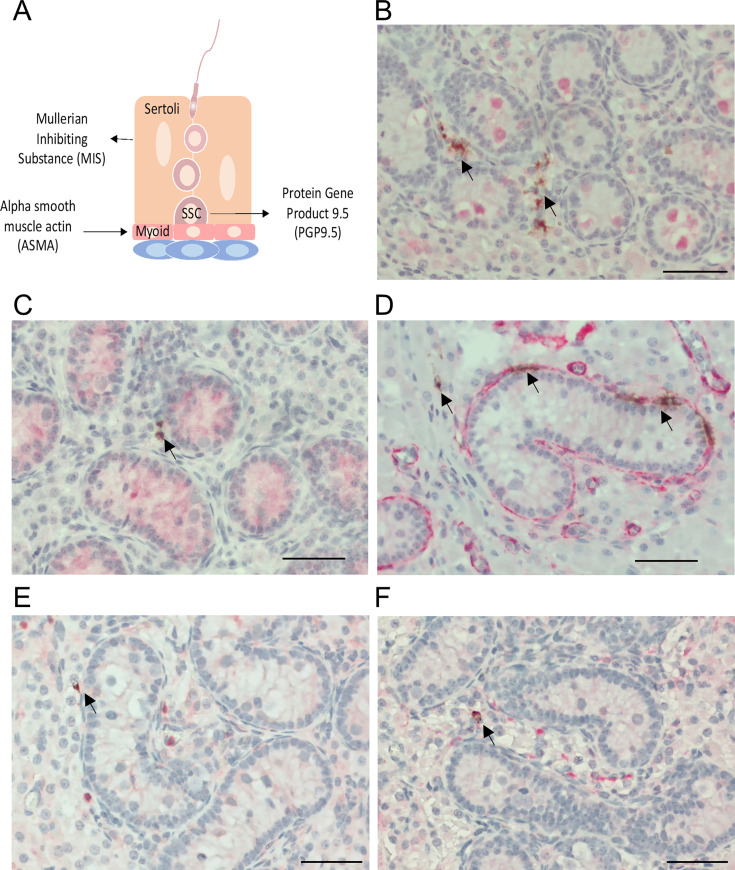
Co-localization of PRRSV mRNA and cellular markers in testicular tissues of PRRSV-infected prepubertal pigs. (**A**) Schematic representation of sexually mature testicular tissue, illustrating cell localization and the specific markers used to identify them. (**B–F**) Sections of testicular tissue were first processed using ISH to detect viral mRNA transcripts with a probe specific to ORF7. Subsequently, the sections were stained by IHC with antibodies specific to various cell markers: PGP9.5 (**B**), MIS (**C**), ASMA (**D**), CD172a (**E**), and CD163 (**F**). Viral mRNA signals are shown in brown, while cellular marker signals are shown in red. Arrows indicate virus-infected cells. Scale bar: 50 µm.

To identify the putative cell types infected by PRRSV in the testes of prepubertal pigs, we performed ISH to detect viral RNA alongside IHC to detect specific cellular markers. We used the following markers to identify different cell types: MIS for Sertoli cells, ASMA for myoid cells, PGP9.5 for SSCs, and CD172a and CD163 for macrophages (Fig. 6A). PGP9.5-positive cells were primarily observed in the center of the seminiferous tubules, separate from the virus-infected cells (Fig. 6B). Likewise, MIS-positive cells were also found within the seminiferous tubules (Fig. 6C) and did not co-localize with the viral RNA signals. ASMA-positive cells were detected surrounding the basement membrane of the seminiferous tubules (Fig. 6D). Notably, ASMA signals co-localized with viral RNA signals, indicating that myoid cells surrounding the seminiferous tubules were infected with PRRSV. On the other hand, cells stained positive for CD172a and CD163 were mainly found in the interstitium, some of which were co-localized with the viral RNA signals (Fig. 6E and F).

## DISCUSSION

We (7) and others (8, 9) have previously reported that PRRSV replicates in the reproductive tissue of sexually active boars, leading to the disruption of spermatogenesis, germ cell depletion, hypospermatogenesis, and the formation of multinucleated giant cells (6, 7). The identification of germ cells being infected with PRRSV was based on the detection of viral RNA within the seminiferous tubules of the testicular tissue (7–9). In this study, we isolated and cultured SSCs from neonatal pigs and demonstrated that these cells are susceptible to PRRSV infection. These results, together with previous reports (7–9), clearly demonstrate the ability of PRRSV to infect cells other than macrophages.

Equine arteritis virus (EAV), a member of the *Arteriviridae* family, can infect stromal and B cells, challenging the notion that arteriviruses have a restricted macrophage tropism (30). Like PRRSV, EAV and lactate dehydrogenase-elevating virus (LDV) are known to persist in male reproductive tissue (31). EAV is also shed in semen and transmitted through the venereal route (32). However, the mechanisms of virus dissemination in the semen seem to be different among arteriviruses. PRRSV is disseminated in the semen in a cell-associated manner, due to its tropism for spermatogonia (7, 33). On the other hand, EAV is disseminated in a cell-free form in seminal plasma, likely due to the release of the virus from infected stromal cells in the ampullae of the stallion’s reproductive tract (30). Viral replication foci have been observed in the seminiferous tubule of mice infected with LDV (31, 34), but demonstrating the presence of the virus in mouse semen is challenging due to the technical difficulties of working with small semen volumes (31).

In this study, SSCs isolated from neonatal pigs and cultured *ex vivo* were highly susceptible to PRRSV infection. The virus replication kinetics in SSCs were similar to those in PAMs, its primary target. However, a notable difference in viral growth was observed between SSCs and MARC-145 cells. While PRRSV replication in SSCs declines after 48 hpi, it remains robust in MARC-145 cells throughout the 72hours observation period. This difference could be attributed to the nature of these cell types. MARC-145 is an established cell line that replicates continuously, while PAMs are primary cells that do not replicate *in vitro*. Similarly, SSCs are primary cells that require feeder cells to grow (35), which were not included when the SSCs were cultured for the growth curve analysis. As a result, the virus has a more abundant supply of cells for replication in MARC-145 than in SSCs and PAMs.

CD163, the major cellular receptor for PRRSV (12), is exclusively expressed by monocytes/macrophages (36, 37). In our IHC analysis, CD163 signals were not found in the SSCs within the seminiferous tubules of prepubertal pigs (Fig. 6F). Thus, CD163 is unlikely to be involved in the susceptibility of SSC to PRRSV infection. Other molecules, including vimentin, CD151, heparan sulfate, sialoadhesin, and DC-SIGN, have been reported to facilitate PRRSV infection (11). Whether these molecules are expressed by SSCs and contribute to their susceptibility to PRRSV infection remains unknown and represents a logical area for further study.

In previous studies, PRRSV infection of SSCs can be detected as early as 7 dpi on sexually mature boars (7). In this study, no PRRSV-infected cells were detected in the seminiferous tubules of prepubertal male pigs at any time point within the 15 days observation period. Instead, virus-infected cells were found in the interstitial tissue, likely originating from macrophages that transport PRRSV into this area. Additionally, PRRSV-infected cells were detected in myoid cells in the periphery of the seminiferous tubules. For viruses with testicular tropism, infection of myoid cells often acts as a conduit to reach other cells within the seminiferous tubules. For instance, Zika virus initially infects cells in the interstitium, then spreads to myoid and Sertoli cells, and eventually reaches the germ cells within the seminiferous tubules (38). Similarly, Marburg virus is transported into the testes by macrophages, subsequently infecting Leydig, myoid, and Sertoli cells, eventually reaching the germinal cells, and the semen (39). In young pigs, SSCs are located in the center of the seminiferous tubules and surrounded by Sertoli cells (40). As pigs reach sexual maturity, SSCs migrate to the base of seminiferous tubules, where they interact with myoid cells (40). We observed that myoid cells at the base of the tubules are susceptible to PRRSV infection. On the other hand, Sertoli cells cultured *ex vivo* are not susceptible to PRRSV infection, suggesting that they may serve as a barrier that prevents the exposure of SSCs to PRRSV. In sexually mature boars, SSCs are in direct contact with myoid cells or peritubular macrophages, which facilitates PRRSV transmission via cell-to-cell contact. In contrast, SSCs in young pigs are surrounded by non-permissive Sertoli cells, which prevent them from being exposed to PRRSV. Thus, the anatomical shift likely explains why SSCs in sexually mature boars are infected with PRRSV, whereas those in young pigs are not.

In summary, we demonstrate that SSCs are highly susceptible to PRRSV infection and provide direct evidence that non-macrophage cells of pigs could be infected with PRRSV. The SSC cultures may serve as a model for investigating alternative viral receptors for PRRSV and offer an excellent platform for developing an *in vitro* model of viral-induced alterations in male spermatogenesis. This model is well suited for integration with advancements in swine testicular organoids (41).

## References

[1] Keffaber K. 1989. Reproductive failure of unknown etiology. Am Assoc Swine Pract Newsl 1:1–9.

[2] Lindhaus W, Lindhaus B. 1991. Mystery swine disease.

[3] Rossow KD. 1998. Porcine reproductive and respiratory syndrome. Vet Pathol 35:1–20. doi:10.1177/030098589803500101 9545131

[4] Prieto C, Suárez P, Bautista JM, Sánchez R, Rillo SM, Simarro I, Solana A, Castro JM. 1996. Semen changes in boars after experimental infection with porcine reproductive and respiratory syndrome (PRRS) virus. Theriogenology 45:383–395. doi:10.1016/0093-691x(95)00375-i 16727802

[5] Christopher-Hennings J, Nelson EA, Hines RJ, Nelson JK, Swenson SL, Zimmerman JJ, Chase CL, Yaeger MJ, Benfield DA. 1995. Persistence of porcine reproductive and respiratory syndrome virus in serum and semen of adult boars. J Vet Diagn Invest 7:456–464. doi:10.1177/104063879500700406 8580165

[6] Prieto C, Castro JM. 2005. Porcine reproductive and respiratory syndrome virus infection in the boar: a review. Theriogenology 63:1–16. doi:10.1016/j.theriogenology.2004.03.018 15589269

[7] Sur JH, Doster AR, Christian JS, Galeota JA, Wills RW, Zimmerman JJ, Osorio FA. 1997. Porcine reproductive and respiratory syndrome virus replicates in testicular germ cells, alters spermatogenesis, and induces germ cell death by apoptosis. J Virol 71:9170–9179. doi:10.1128/JVI.71.12.9170-9179.1997 9371575 PMC230219

[8] Han K, Seo HW, Park C, Oh Y, Kang I, Chae C. 2013. Comparative pathogenesis of type 1 (European genotype) and type 2 (North American genotype) porcine reproductive and respiratory syndrome virus in infected boar. Virol J 10:156. doi:10.1186/1743-422X-10-156 23687995 PMC3663669

[9] Han K, Seo HW, Oh Y, Kang I, Park C, Han JH, Kim SH, Chae C. 2013. Pathogenesis of type 1 (European genotype) porcine reproductive and respiratory syndrome virus in male gonads of infected boar. Vet Res Commun 37:155–162. doi:10.1007/s11259-013-9558-x 23435841

[10] Christopher-Hennings J, Nelson EA, Nelson JK, Rossow KD, Shivers JL, Yaeger MJ, Chase CC, Garduno RA, Collins JE, Benfield DA. 1998. Identification of porcine reproductive and respiratory syndrome virus in semen and tissues from vasectomized and nonvasectomized boars. Vet Pathol 35:260–267. doi:10.1177/030098589803500404 9684969

[11] Zhang Q, Yoo D. 2015. PRRS virus receptors and their role for pathogenesis. Vet Microbiol 177:229–241. doi:10.1016/j.vetmic.2015.04.002 25912022

[12] Calvert JG, Slade DE, Shields SL, Jolie R, Mannan RM, Ankenbauer RG, Welch S-KW. 2007. CD163 expression confers susceptibility to porcine reproductive and respiratory syndrome viruses. J Virol 81:7371–7379. doi:10.1128/JVI.00513-07 17494075 PMC1933360

[13] Whitworth KM, Rowland RRR, Ewen CL, Trible BR, Kerrigan MA, Cino-Ozuna AG, Samuel MS, Lightner JE, McLaren DG, Mileham AJ, Wells KD, Prather RS. 2016. Gene-edited pigs are protected from porcine reproductive and respiratory syndrome virus. Nat Biotechnol 34:20–22. doi:10.1038/nbt.3434 26641533

[14] Yang H, Zhang J, Zhang X, Shi J, Pan Y, Zhou R, Li G, Li Z, Cai G, Wu Z. 2018. CD163 knockout pigs are fully resistant to highly pathogenic porcine reproductive and respiratory syndrome virus. Antiviral Res 151:63–70. doi:10.1016/j.antiviral.2018.01.004 29337166

[15] Burkard C, Opriessnig T, Mileham AJ, Stadejek T, Ait-Ali T, Lillico SG, Whitelaw CBA, Archibald AL. 2018. Pigs lacking the scavenger receptor cysteine-rich domain 5 of CD163 are resistant to porcine reproductive and respiratory syndrome virus 1 infection. J Virol 92:00415–00418. doi:10.1128/JVI.00415-18 PMC6069206 29925651

[16] Salgado B, Rivas RB, Pinto D, Sonstegard TS, Carlson DF, Martins K, Bostrom JR, Sinebo Y, Rowland RRR, Brandariz-Nuñez A. 2024. Genetically modified pigs lacking CD163 PSTII-domain-coding exon 13 are completely resistant to PRRSV infection. Antiviral Res 221:105793. doi:10.1016/j.antiviral.2024.105793 38184111

[17] Kim JK, Fahad AM, Shanmukhappa K, Kapil S. 2006. Defining the cellular target(s) of porcine reproductive and respiratory syndrome virus blocking monoclonal antibody 7G10. J Virol 80:689–696. doi:10.1128/JVI.80.2.689-696.2006 16378972 PMC1346842

[18] Shanmukhappa K, Kim J-K, Kapil S. 2007. Role of CD151, a tetraspanin, in porcine reproductive and respiratory syndrome virus infection. Virol J 4:62. doi:10.1186/1743-422X-4-62 17572908 PMC1906853

[19] Kim HS, Kwang J, Yoon IJ, Joo HS, Frey ML. 1993. Enhanced replication of porcine reproductive and respiratory syndrome (PRRS) virus in a homogeneous subpopulation of MA-104 cell line. Arch Virol 133:477–483. doi:10.1007/BF01313785 8257302

[20] Truong HM, Lu Z, Kutish GF, Galeota J, Osorio FA, Pattnaik AK. 2004. A highly pathogenic porcine reproductive and respiratory syndrome virus generated from an infectious cDNA clone retains the in vivo virulence and transmissibility properties of the parental virus. Virology (Auckl) 325:308–319. doi:10.1016/j.virol.2004.04.046 PMC7127741 15246270

[21] Park MH, Park JE, Kim MS, Lee KY, Park HJ, Yun JI, Choi JH, Lee E song, Lee ST. 2014. Development of a high-yield technique to isolate spermatogonial stem cells from porcine testes. J Assist Reprod Genet 31:983–991. doi:10.1007/s10815-014-0271-7 24938360 PMC4130942

[22] Chiappalupi S, Luca G, Mancuso F, Madaro L, Fallarino F, Nicoletti C, Calvitti M, Arato I, Falabella G, Salvadori L, Di Meo A, Bufalari A, Giovagnoli S, Calafiore R, Donato R, Sorci G. 2016. Intraperitoneal injection of microencapsulated Sertoli cells restores muscle morphology and performance in dystrophic mice. Biomaterials 75:313–326. doi:10.1016/j.biomaterials.2015.10.029 26523508

[23] Chaudhari J, Leme RA, Durazo-Martinez K, Sillman S, Workman AM, Vu HLX. 2022. A single amino acid substitution in porcine reproductive and respiratory syndrome virus glycoprotein 2 significantly impairs its infectivity in macrophages. Viruses 14:2822. doi:10.3390/v14122822 36560826 PMC9781675

[24] Ryu B-Y, Kubota H, Avarbock MR, Brinster RL. 2005. Conservation of spermatogonial stem cell self-renewal signaling between mouse and rat. Proc Natl Acad Sci USA 102:14302–14307. doi:10.1073/pnas.0506970102 16183739 PMC1242328

[25] Zhang P, Chen X, Zheng Y, Zhu J, Qin Y, Lv Y, Zeng W. 2017. Long-term propagation of porcine undifferentiated spermatogonia. Stem Cells Dev 26:1121–1131. doi:10.1089/scd.2017.0018 28474535 PMC5563923

[26] Kanatsu-Shinohara M, Muneto T, Lee J, Takenaka M, Chuma S, Nakatsuji N, Horiuchi T, Shinohara T. 2008. Long-term culture of male germline stem cells from hamster testes. Biol Reprod 78:611–617. doi:10.1095/biolreprod.107.065615 18094355

[27] Štefková K, Procházková J, Pacherník J. 2015. Alkaline phosphatase in stem cells. Stem Cells Int 2015:628368. doi:10.1155/2015/628368 25767512 PMC4342173

[28] Luo J, Megee S, Rathi R, Dobrinski I. 2006. Protein gene product 9.5 is a spermatogonia-specific marker in the pig testis: application to enrichment and culture of porcine spermatogonia. Mol Reprod Dev 73:1531–1540. doi:10.1002/mrd.20529 16894537

[29] Duan X, Nauwynck HJ, Pensaert MB. 1997. Virus quantification and identification of cellular targets in the lungs and lymphoid tissues of pigs at different time intervals after inoculation with porcine reproductive and respiratory syndrome virus (PRRSV). Vet Microbiol 56:9–19. doi:10.1016/S0378-1135(96)01347-8 9228678

[30] Carossino M, Loynachan AT, Canisso IF, Cook RF, Campos JR, Nam B, Go YY, Squires EL, Troedsson MHT, Swerczek T, Del Piero F, Bailey E, Timoney PJ, Balasuriya UBR. 2017. Equine arteritis virus has specific tropism for stromal cells and CD8^+^ T and CD21^+^ B lymphocytes but not for glandular epithelium at the primary site of persistent infection in the stallion reproductive tract. J Virol 91:00418–00517. doi:10.1128/JVI.00418-17 PMC5469258 28424285

[31] Anderson GW, Rowland RR, Palmer GA, Even C, Plagemann PG. 1995. Lactate dehydrogenase-elevating virus replication persists in liver, spleen, lymph node, and testis tissues and results in accumulation of viral RNA in germinal centers, concomitant with polyclonal activation of B cells. J Virol 69:5177–5185. doi:10.1128/JVI.69.8.5177-5185.1995 7609091 PMC189342

[32] Glaser AL, Chirnside ED, Horzinek MC, de Vries AA. 1997. Equine arteritis virus. Theriogenology 47:1275–1295. doi:10.1016/s0093-691x(97)00107-6 16728076 PMC7127492

[33] Christopher-Hennings J, Nelson EA, Nelson JK, Hines RJ, Swenson SL, Hill HT, Zimmerman JJ, Katz JB, Yaeger MJ, Chase CC. 1995. Detection of porcine reproductive and respiratory syndrome virus in boar semen by PCR. J Clin Microbiol 33:1730–1734. doi:10.1128/jcm.33.7.1730-1734.1995 7665637 PMC228258

[34] Plagemann PG, Rowland RR, Even C, Faaberg KS. 1995. Lactate dehydrogenase-elevating virus: an ideal persistent virus? Springer Semin Immunopathol 17:167–186. doi:10.1007/BF00196164 8571167 PMC7087530

[35] Brinster RL, Avarbock MR. 1994. Germline transmission of donor haplotype following spermatogonial transplantation. Proc Natl Acad Sci USA 91:11303–11307. doi:10.1073/pnas.91.24.11303 7972054 PMC45219

[36] Pulford K, Micklem K, McCarthy S, Cordell J, Jones M, Mason DY. 1992. A monocyte/macrophage antigen recognized by the four antibodies GHI/61, Ber-MAC3, Ki-M8 and SM4. Immunology 75:588–595. 1592433 PMC1384835

[37] Kristiansen M, Graversen JH, Jacobsen C, Sonne O, Hoffman HJ, Law SK, Moestrup SK. 2001. Identification of the haemoglobin scavenger receptor. Nature 409:198–201. doi:10.1038/35051594 11196644

[38] Matusali G, Houzet L, Satie AP, Mahé D, Aubry F, Couderc T, Frouard J, Bourgeau S, Bensalah K, Lavoué S, Joguet G, Bujan L, Cabié A, Avelar G, Lecuit M, Le Tortorec A, Dejucq-Rainsford N. 2018. Zika virus infects human testicular tissue and germ cells. J Clin Invest 128:4697–4710. doi:10.1172/JCI121735 30063220 PMC6159993

[39] Coffin KM, Liu J, Warren TK, Blancett CD, Kuehl KA, Nichols DK, Bearss JJ, Schellhase CW, Retterer CJ, Weidner JM, Radoshitzky SR, Brannan JM, Cardile AP, Dye JM, Palacios G, Sun MG, Kuhn JH, Bavari S, Zeng X. 2018. Persistent marburg virus infection in the testes of nonhuman primate survivors. Cell Host Microbe 24:405–416. doi:10.1016/j.chom.2018.08.003 30173956

[40] Zheng Y, Gao Q, Li T, Liu R, Cheng Z, Guo M, Xiao J, Wu D, Zeng W. 2022. Sertoli cell and spermatogonial development in pigs. J Anim Sci Biotechnol 13:45. doi:10.1186/s40104-022-00687-2 35399096 PMC8996595

[41] Sakib S, Uchida A, Valenzuela-Leon P, Yu Y, Valli-Pulaski H, Orwig K, Ungrin M, Dobrinski I. 2019. Formation of organotypic testicular organoids in microwell culture†. Biol Reprod 100:1648–1660. doi:10.1093/biolre/ioz053 30927418 PMC7302515

